# Phosphate Ion Release and Alkalizing Potential of Three Bioactive Dental Materials in Comparison with Composite Resin

**DOI:** 10.1155/2021/5572569

**Published:** 2021-05-07

**Authors:** Shahin Kasraei, Sahebeh Haghi, Sara Valizadeh, Narges Panahandeh, Sogol Nejadkarimi

**Affiliations:** ^1^Department of Restorative Dentistry, Dental School, Shahid Beheshti University of Medical Sciences, Tehran, Iran; ^2^Department of Restorative Dentistry, Dental School, Arak University of Medical Sciences, Arak, Iran; ^3^Dental Research Center, Dentistry Research Institute, Restorative Dentistry Department, Dental School, Tehran University of Medical Sciences, Tehran, Iran

## Abstract

**Aim:**

Several new bioactive compounds were recently introduced to the market with favorable ion release, tooth remineralization, and alkalizing potential. This study sought to compare the phosphate ion release and alkalizing potential of three bioactive materials in comparison with composite resin.

**Methods:**

Thirty-six discs (2 × 6 mm) were fabricated from Fuji II LC resin modified glass ionomer (RMGI), Activa BioActive, Cention N, and Z250 composite in plastic molds. The specimens were stored in distilled water for 24 and 48 h and 6 months. Half of the specimens were used to assess the phosphate ion release while the other half were used to assess the alkalizing potential 1 h after pH drop from 6.8 to 4. Phosphate ion release was quantified by a spectrophotometer while the pH value was measured by a pH meter. Data were analyzed using two-way ANOVA, one-way ANOVA, and Tukey's HSD test (for pairwise comparisons) at 0.05 level of significance.

**Results:**

At 24 h, the maximum phosphate ion release in distilled water occurred in the Fuji II LC group followed by Cention N, Activa BioActive, and Z250. At 6 months, Cention N followed by Activa BioActive showed higher phosphate ion release than Fuji II LC and Z250. No significant difference was noted between Activa BioActive and Cention N at any time point. All materials, except for Z250, increased the pH of the environment. Fuji II LC had maximum alkalizing effect at all time points followed by Cention N and Activa BioActive.

**Conclusion:**

Use of bioactive compounds is a promising method to ensure phosphate ion release, and can have a positive effect on tooth remineralization over time. Also, bioactive compounds can alkalize an acidic environment.

## 1. Introduction

Advances in dental material technology enabled the use of direct tooth-colored restorative materials for dental restorations [1]. Dental composites have been used for over 40 years for restoration of the lost tooth structure. However, they have no significant effect on the cariogenic bacteria and have no positive effect on the ionic balance of the adjacent tooth structure. Moreover, some reports are available regarding enhanced cariogenic biofilm growth next to composite restorations [2, 3]. These parameters can increase the risk of bacterial microleakage at the tooth-composite interface and development of secondary caries, which is the main cause of failure of composite restorations [4]. According to a previous study, one-fifth of dental patients experience at least one secondary caries in their oral cavity [5]. Another study reported a high rate of development of secondary caries around composite restorations, estimated at 60% [6].

Despite the great improvements in the mechanical and esthetic properties of composite resins in the past two decades, attempts are ongoing to find methods to stop the development of secondary caries beneath and around the restorative materials. However, the majority of the available composite resins in the market have no remineralizing effect [7]. This can be due to the fact that most dental restorative materials are neutral and do not trigger any physiological response in the host tissue. They only fill the empty spaces and cavities following the loss of tooth structure and cannot prevent subsequent complications such as development of recurrent caries due to acidic oral environment [8]. In order to overcome such problems, there is an increasing tendency to use resin-based bioactive and remineralizing restorative materials to increase the durability of bonded restorations. By doing so, rate of caries at the restoration margins may decrease as a result of interactions of these materials in the oral environment and enhancement of remineralization, releasing different ions, increasing the pH, and inducing the deposition of hydroxyapatite [9].

Release of different remineralizing ions from the restorative materials into the oral cavity and at the tooth-restoration interface improves the longevity of restorations and decreases the rate of recurrent caries. For instance, release of fluoride, calcium, and phosphate ions that play a role in tooth remineralization seems to be an imperative property for the future restorative materials [10]. These ions can eliminate the microorganisms and cause deposition of apatite-like compounds at the tooth-restoration interface, which would increase the tooth resistance to acid attacks and prolong the clinical service of restorations in the oral cavity. Moreover, these materials can respond to pH alterations in the oral cavity by uptake or release of calcium, phosphorous, and fluoride ions and preserve the integrity of tooth structure as such [11].

The ability to react to environmental changes is another property of bioactive materials. In case of pH drop in the oral environment, bioactive materials can release hydroxyl ions to neutralize the acid produced by the biofilm and alkalize the environment. This would cause elimination of bacteria, prevent tooth demineralization, and enhance remineralization [12].

After silicate cements, glass ionomers are among the first reactive tooth-colored restorative materials that can release fluoride and have the recharge potential. Glass ionomers are commonly used in dentistry due to favorable characteristics such as inherent adhesion to tooth structure, having a coefficient of thermal expansion similar to that of tooth, and high biocompatibility [13]. Nonetheless, they have limitations such as high wear, high solubility, poor mechanical properties, and low strength against occlusal forces. For instance, compressive strength of RMGIs is between 150 and 166 MPa compared to composite resins in the range of 265–290 MPa [14].

Activa BioActive is a tooth-colored bioactive restorative material with strong mechanical properties, which was recently introduced to the market. Unlike composite resins, it does not contain bisphenol A or its derivatives. Thus, it has higher biocompatibility [15]. It is a novel type of composite, which is a claim of bioactive material with reviving hydroxyapatite layer formation and natural remineralization at the tooth-restoration interface by significant release and recharge of calcium, phosphorus, and fluoride ions and also has acceptable esthetic properties. It also possesses all the advantages of glass ionomer cements in a strong and flexible resin matrix. It contains bioactive glass and a hydrophilic rubbery resin matrix, which can release calcium, phosphate, and fluoride without compromising its physical properties [16].

Cention N is another tooth-colored bioactive restorative material with high flexural strength. It is an alkasite dental material, which can be used as bulk for restoration of teeth [17]. Cention N is supplied in the form of powder and liquid, and the manufacturer claims that it serves as an alternative to amalgam. This dual-cure restorative material has alkaline fillers embedded methacrylate resin matrix and releases hydroxyl ions; thus, it can neutralize the activity of acidogenic cariogenic bacteria [18].

Considering the passivity of dental composites and the fact that they have no role in protection of restored teeth against secondary caries, use of new bioactive restorative materials may help decrease the incidence of secondary caries and increase the success rate of restorative treatments due to their ion release, tooth remineralization, and alkalizing potential. This study sought to compare the release of phosphate ions and alkalizing potential of three bioactive materials, in comparison with composite resin.

## 2. Materials and Methods

A total of 36 discs measuring 6 mm in diameter and 2 mm in thickness were fabricated from each restorative material, Activa BioActive, Cention N, and Fuji II LC, Z250, (a total of 144 specimens) using plastic molds. Materials used in this study and their composition are shown in Table 1. The molds were placed on a glass slab and the restorative materials were applied into the molds according to the manufacturers' instructions and condensed. They were then covered with a transparent Mylar strip and a glass slab was placed over them. The specimens were cured with a light-curing unit (DRS Light AT; Good Doctors Co., Ltd., Korea) with a light intensity of 1200 mW/cm^2^ through the glass slab. The intensity of light was measured after curing 5 specimens using a radiometer (LM-1 Light Meter, Woodpecker, Guilin, China). The specimens were then removed from the molds and polished with 600- and 800-grit abrasive papers under running water. Half of the specimens were used for quantification of the released phosphate ions while the other half were used to measure the hydroxyl ions released from the restorative materials.

### 2.1. Measuring the Alterations in Phosphate Ion Concentration

The cumulative amount of phosphate ions released into a neutral aqueous environment with a pH of 6.8 and an acidic environment with a pH of 4 from each restorative material (half of the specimens, *n* = 72) was measured after 24 h, 48 h, and 6 months of storage in aqueous solutions and reported in micrograms/square centimeters (*µ*g/cm^2^).

Eighteen specimens fabricated from each restorative material were randomly divided into three subgroups (*n* = 6) for storage for 24 h, 48 h, and 6 months. Each specimen was placed in a container containing 5 mL of distilled water with a pH of 6.8 at 37°C. The pH of the solution in the container was measured by a pH meter (Metrohm 744, Metrohm Ltd., Herisa, Switzerland), which had been previously calibrated with pH values of 4, 7, and 10.

After immersion of specimens in distilled water with a pH of 6.8 for 24 h, 1 mL of the solution was removed from each of the first containers (*n* = 3) in the three subgroups by a micropipette and diluted with 9 mL of distilled water. Next, one unit of phosphate reagent (Cat 21060-69) was added to the solution and well shaken for 20–30 s to obtain a homogenous mixture. The solution then underwent spectrophotometry (DR-5000, HACH Co., Loveland, USA) at a wavelength of 980 nm to determine the cumulative amount of released phosphate ions into the solution (pH = 6.8) at 24 h in milligrams/liter (mg/L) [19].

Next, 50 mmol/L lactic acid was added to each of the second three screw-top containers in the three subgroups to decrease the pH from 6.8 to 4. The specimens were stored in this pH at 37°C for 1 h. Next, 1 mL of the contents of each container was collected with a pipette and diluted with 9 mL of distilled water. The amount of phosphate ions released into the solution was quantified by spectroscopy and reported in milligrams/liter (mg/L). The data were then converted to micrograms/square centimeters (*µ*g/cm^2^) using the formula below to increase their applicability to the clinical setting and determine the effect of specimen surface area on ion release and for the purpose of comparison with previous studies.

(1) $$\begin{array}{c} \text{Ion release}=\frac{\mu g}{ml}\times \left(\frac{\text{volume of solution}}{\text{surface area of sample}}\right)\left(\text{Surface area }\left({\text{cm}}^{2}\right)\right)=2\pi r\left(r+h\right). \end{array}$$

The same procedure was repeated for all six specimens in the second (48 h) and third (6 months) subgroups of each restorative material to measure the cumulative amount of phosphate ions released into the solution. The only difference was the storage time of specimens, which was 48 h for the second subgroup and 6 months for the third subgroup.

### 2.2. Determining the Alkalizing Potential of Materials

The alkalizing potential of each restorative material was determined based on their ability to increase the pH of the storage solution after its acidification. Eighteen specimens fabricated from each restorative material (a total of 72 specimens) were separately placed in screw-top containers containing 5 mL of distilled water with a pH of 6.8 at 37°C. The pH alterations after immersion of specimens in a neutral aqueous environment (pH = 6.8) and acidic environment (pH = 4) were studied separately for each restorative material after 24 h, 48 h, and 6 months of storage in distilled water. The distribution of specimens in the subgroups for measurement of alkalizing potential was similar to that for measurement of phosphate ion release, with the difference that the measurements were made at the respective time points following addition of lactic acid and subsequent pH reduction of the storage solution from 6.8 to 4. The pH of the solution was repeatedly measured within one hour at 0, 10, 20, 30, 40, 50, and 60 min, and recorded. The graph of pH alterations was also drawn. It should be noted that only the pH alteration data at 0 and 60 min were statistically analyzed at 0.05 level of significance.

### 2.3. Statistical Analysis

Data regarding the amount of released phosphate ions before and after immersion in acidic solution were compared among the four restorative materials and the three time points of 24 h, 48 h, and 6 months using two-way ANOVA, one-way ANOVA, and Tukey's HSD post hoc test at 0.05 level of significance. The pH alteration data were similarly analyzed at 0.05 level of significance.

## 3. Results

### 3.1. Phosphate Ion Release

Two-way ANOVA showed that type of restorative material (*P*=0.001) and duration of storage in distilled water (*P*=0.001) had significant effects on the amount of ions released from the restorative materials following pH reduction from 6.8 to 4. The interaction effect of duration of storage and type of restorative material was also significant (*P*=0.001). Thus, data were analyzed by one-way ANOVA and Tukey's HSD test at each time point. The results showed maximum release of phosphate ions at 1 h after pH reduction of the solution containing Fuji II LC glass ionomer specimens stored for 24 h (*P*=0.001). Z250 composite specimens showed minimum release of phosphate ions at all time points (*P* < 0.001). Also, no change occurred in the release of phosphate ions from composite specimens over time (*P*=0.38).  Table 2 shows the mean and standard deviation of the amount of phosphate ions released from the restorative materials at the three time points.

The phosphate ion release from Activa BioActive and Cention N bioactive materials significantly increased as the storage time increased from 24 h to 48 h, and 6 months in distilled water following a reduction in pH from 6.8 to 4 (*P*=0.001). However, the change in ion release from Fuji II LC under similar conditions was not significant (*P*=0.052).

**Figure 1:**
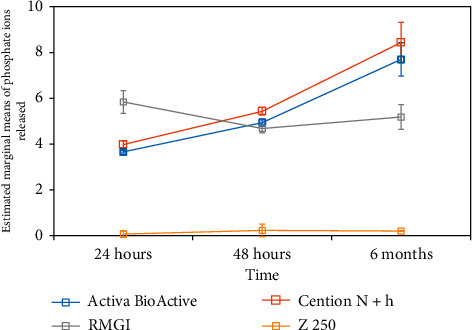
Comparison of the mean values change in phosphate ion release (*μ*g/cm^2^) from the stored restorative materials following pH reduction from 6.8 to 4 at different time points.

Pairwise comparisons of the change in ion release following pH reduction at 24 h, 48 h, and 6 months revealed no significant difference between the Activa BioActive and Cention N (*P*=0.074, *P*=0.052, and *P*=0.552, respectively). In brief, the order of minimum ion release following pH reduction after 24 h of storage in distilled water was as follows: Z250 < Activa BioActive = Cention *N* < Fuji II LC. This order was Z250 < Fuji II LC < Activa BioActive = Cention NZ250 after 6 months.  Figure 1 compares the mean change in phosphate ion release (*μ*g/cm^2^) from the restorative materials upon increasing the acidity of the solution after storage for 24 h, 48 h, and 6 months in distilled water.

### 3.2. Alkalizing Potential

**Figure 2:**
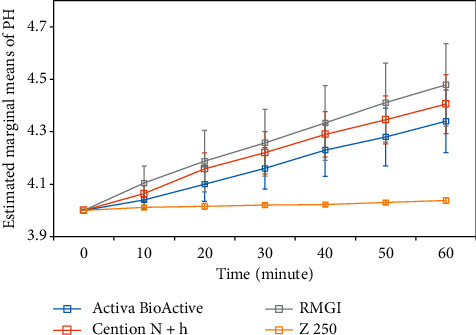
pH value measured at 0, 10, 20, 30, 40, 50, and 60 min following acidification of the environment from pH 6.8 to 4.

The alkalizing potential of restorative materials was measured after pH reduction from 6.8 to 4 by quantifying the positive change in the pH at different time points.  Figure 2 shows the pH value of different restorative materials after acidifying the environment (pH = 4) at 0, 10, 20, 30, 40, 50, and 60 min.

Table 3 shows the mean increase in the pH of solution, indicating the release of hydroxyl ions and the alkalizing potential of restorative materials after 1 h of acidification of the solution (pH = 4). Repeated measures two-way ANOVA revealed that type of restorative material and duration of storage of specimens in distilled water had significant effects on pH alterations within one hour (*P*=0.001 and *P*=0.001, respectively). The interaction effect of the two factors was also significant (*P*=0.001). Thus, data were analyzed using one-way ANOVA and Tukey's HSD test at each time point (Table 3).

**Figure 3:**
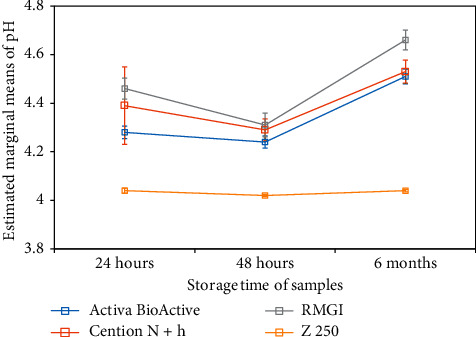
Mean changes in pH value after acidification of the medium (pH = 4) and placing samples (24 hours, 48 hours, and 6 months) in it for 60 minutes.

As shown in Table 3, in all restorative material groups except for Z250 composite, the pH of the environment increased over time following pH reduction from 6.8 to 4. The alkalizing potential of restorative materials was variable at different time points (*P*=0.001). The difference in pH increase was not significant at 24 and 48 h (*P* > 0.05). However, the difference in pH increase was significant at 6 months (*P*=0.001) such that the minimum change in pH was noted at 48 h while the maximum pH increase was noted at 6 months. At all time points, Fuji II LC had the maximum alkalizing effect and caused the maximum pH increase in acidic environment followed by Cention N and Activa BioActive.  Figure 3 shows the positive changes in pH after acidification of the environment (pH = 4) at different time points.

## 4. Discussion

Application of restorative materials that cause a local increase in calcium and phosphate ions and have the potential to alkalize the oral environment is a promising method to prevent secondary caries and progression of carious lesions [20]. This study compared the amount of released phosphate ions and the alkalizing potential of three bioactive materials compared with composite resin.

Phosphate ions play a role in formation and deposition of hydroxyapatite on the tooth surface and structures adjacent to a restoration. They also have a prominent role in the process of remineralization [21].

In this study, the amount of released phosphate ions at 1 h after pH reduction was significantly greater in Fuji II LC resin modified glass ionomer (RMGI) group stored for 24 h. This can be due to the high-water sorption at the onset of acid-base reactions and high speed of reactions, causing the release of higher amounts of phosphate ions compared with other materials [22].

Mazzaoui et al. showed that burst release of ions in the first 24 h of storage of RMGI is due to the superficial dissolution of this material. However, this dissolution decelerates after a while. Consequently, ion release from the mass material into the environment decreases [23]. On the other hand, presence of HEMA in the composition of Fuji II LC can be another reason for its higher water sorption at the onset of reactions [24].

At 24 h, Cention N and Activa BioActive released smaller amounts of phosphate ions than RMGI, which can be due to the formation of a superficial layer of calcium fluoride and calcium phosphate with 0.5 mm thickness on the surface of Cention N at the initiation of setting reactions, which resists dissolution for some time [25]. Garoushi et al. showed that release of ions from RMGI in the first 24 h was higher than that from Activa BioActive, which was in line with our findings [26]. The resin structure of Activa BioActive and its lower dissolution explain this finding.

Greater release of phosphate ions from Cention N compared with Activa BioActive at 24 h can be related to the formation of voids and porosities in Cention N during mixing of powder and liquid, and presence of alkaline fillers of calcium fluorosilicate. Although this change was not statistically significant, presence of voids leads to water sorption and dissolution of material [27]. Moreover, presence of voids prevents the polymerization reactions and increases the amount of unpolymerized material and leads to further ion release [28]. Activa BioActive is a resin-based paste; thus, its lower ion release potential compared with Cention N is somehow expected [29]. By an increase in duration of storage of specimens from 24 h to 48 h and 6 months, Cention N followed by Activa BioActive (with no significant difference) and then Fuji II LC (with significant differences with Cention N and Activa BioActive) showed higher release of phosphate ions following acidification of the environment. The manufacturers of Activa BioActive and Cention N claim that they have higher ion release potential than RMGI and conventional glass ionomers [30].

In this study, phosphate ion release from Cention N and then Activa BioActive increased by increasing the duration of storage to 6 months, and this increase was greater than that in Fuji II LC. This finding was in contrast to the results of Garoushi et al., who reported that Fuji II LC had maximum phosphate ion release compared with two compomers, one giomer, and Activa BioActive [26]. However, in their study, ion release was evaluated for 10 days, and different results could have been obtained in longer periods of time (6 months). A previous study showed that Cention N does not have an initial burst, as does the glass ionomer. However, it releases higher amounts of ions over time, which may be due to its higher powder/liquid ratio and the amount of alkaline glass in the final stage [31]. Cention N contains three types of inorganic glasses, namely, a conventional barium aluminosilicate glass, a glass ionomer with calcium barium fluoroaluminosilicate glass base, and alkaline fluorosilicate glass, known as alkasite fillers [32].

Detailed information is not available regarding the composition of Activa BioActive glass. Garoushi et al. stated that Activa BioActive probably contains bioactive glass [26]. Dissolution of bioglass occurs by the cleavage of Si-O-Si bonds, which rapidly increases the concentration of phosphate ions, and can lead to formation of apatite or similar compounds. O'Donnel et al. [33] showed that the amount and speed of formation of apatite increased by an increase in bioactive phosphate glass content. Porenczuk et al. reported that ion release from Activa BioActive after 14 days was less than that from glass ionomer and higher than that from composite. However, over time, glass ionomer specimens showed a greater reduction in ion release than Activa BioActive; their results were in line with the current findings [34].

Z250 composite specimens showed the least phosphate ion release among the tested materials at all time points in this study. Also, no change in phosphate ion release of composite specimens was noted over time. This finding can be due to the relatively higher stability of the matrix structure and fillers of composite resins against acid attacks, and absence of glass matrix and bioactive components in its composition.

In all restorative materials, except for Z250 composite, the pH rise following pH drop from 6.8 to 4 improved over time as their duration of storage increased. Czarnecka et al. indicated that RMGIs can neutralize the pH of their storage medium by the release of different ions. Ion release from glass ionomers occurs following acidic hydrolysis of Al-O-Si bonds. Ion release occurs soon after immersion of materials in acid due to water sorption; but over time, it occurs due to erosion [35]. However, ion release from RMGI does not have a direct correlation with the pH of the environment and may even occur in a neutral pH. However, it occurs at a faster pace in an acidic environment [36].

The alkaline glass fillers of Cention N can release hydroxyl ions, increase the pH of the environment, and neutralize the acidic environment caused by the activity of cariogenic bacteria when placed in an acidic environment with a subcritical pH. It appears than ionic exchange of Ca^2+^ with H^+^ plays a role in this process and is responsible for this property of Cention N [18]. Alkasite materials contain alkaline fillers that release acid-neutralizing ions. In the mixed form of Cention N, the weight percentage of alkaline glass is around 24.6%, which is responsible for ion release [37].

The first step of Activa reaction includes the exchange of Ca2^+^ and Na^+^ ions with H^+^, which increases the pH of the environment. Presence of HEMA and TEGDMA can cause hydrolytic degradation of bioglass particles [38]. However, as mentioned earlier, no information has been disclosed regarding the type of glass used in its composition.

Ion release increases with a reduction in pH and longer duration of water storage of specimens until it reaches a plateau due to increased surface area of the particles following their further dissolution [23].

In this study, Fuji II LC showed maximum alkalizing effect at all time points followed by Cention N and Activa Bioactive. In total, higher solubility of Fuji II LC and presence of poly-HEMA hydrogel phase in its composition cause further water sorption and greater release of hydroxyl ions. However, increased ion release over time leads to degradation and compromises the mechanical properties of the material. Moreover, the buffering effect of RMGI is also reinforced by the leaching of aluminum ions into the acidic environment and can also play a role in pH rise following acidification [21].

Lower release of hydroxyl ions and lower alkalizing potential of Cention N compared with Fuji II LC can be due to surface modification of Cention fillers, making them more resistant to degradation. Thus, during immersion in distilled water, they show lower rate of hydroxyl ion release. Also, it does not contain HEMA, or TEGDMA, which are both hydrophilic and have high solubility [32]. Activa BioActive also released lower rate of hydroxyl ions due to its polymerized mesh network compared with Fuji II LC and Cention N, although the difference between Activa BioActive and Cention N did not reach statistical significance [38].

Gupta et al. evaluated the hydroxyl ion release from Cention N and glass ionomer immersed in distilled water and acidic solution and concluded that Cention N was more capable of neutralizing an acidic environment [34]. Their results were in contrast to our findings. However, they evaluated Cention N in self-cure and light-cure forms. Self-cure Cention N had a significantly greater alkalizing potential than the light-cure Cention N and RMGI. This finding can be due to its lower degree of conversion when polymerized as self-cure and its subsequently greater solubility and ion release.

The current results indicated a significant increase in the pH following acidification of the environment. Nonetheless, it seems that increasing the alkalinity of the environment (pH change by 0.5 to 0.6) is not great enough to prevent demineralization. Since the pH of the environment was still below the critical threshold, the process of demineralization probably continues clinically, although it may decelerate [39]. Clinical studies are required to further elucidate this topic.

It should be noted that, due to the absence of the flushing effect and buffering capacity of the saliva, the effect of acid is often overestimated in vitro. In oral physiological conditions, it takes around 30 min for the saliva to neutralize the acid produced by the biofilm. Application of bioactive agents can shorten the demineralization time and have a protective effect on the tooth structure. Eventually, by an increase in concentrations of calcium, phosphorous, and fluoride adjacent to the tooth structure and benefitting from their alkalizing potential, the equilibrium can change towards remineralization even in acidic conditions [40].

## 5. Conclusion

Within the limitations of this study, it may be concluded that, upon pH reduction and acidification of the environment, the amount of released phosphate ions from all three tested restorative materials significantly increased compared with composite resin. The ion release was maximum from RMGI at 24 h, and Cention N and Activa BioActive at 6 months. All three restorative materials increased the pH of the environment, compared with composite resin. The alkalizing potential of RMGI was higher than that of Cention N and Activa BioActive at all time points.

## Figures and Tables

**Table 1:** Materials and their composition used in this study.

Materials	Manufacturer	Composition	Filler
Activa bioactive, ionic composite resin	Pulpdent, Corporation, Watertown, MA, USA	Blend of diurethane and other methacrylates with modified poly acrylic acid (44.6 %), contain no bisphenol A, noBis GMA, no BPA derivatives	Amorphous silica( 6.7%), Sodium fluoride (0.75%)

Cention N, alkasite, restorative, material	Ivoclar-Vivadent, Liechtenstein, Switzerland	UDMA, No Bis GMA, No TEGDMA, No HEMA	Barium aluminum silicate glass, Ytterbium trifluoride, Iso filler, calcium barium aluminum fluorosilicate glass, calcium fluorosilicate glass (57.6%)

Filtek Z250, Composite resin	3M ESPE, St, Paul, MN, USA	Bis GMA, UDMA, Bis EMA	Zirconia/silica

Fuji II LC, Capsulated, resin modified glassionomer cement	GC Corporation, Tokyo, Japan	Alumino-fluorosilicate glass, polyacrylic acid, 2-hydroxyethylmethacrylate, 2,2,4-trimethyl hexamethylene dicarbonate, triethylene glycol, Dimethacrylate	55 vol% FSG /poly-HEMA, average 5.9 *μ*m

**Table 2:** Mean (and standard deviation) amount of phosphate ions released (*µ*g/cm^2^) from restorative materials following pH reduction from 6.8 to 4 at different time points of storage.

Materials	24 hours	^*∗*^ *P*-value	48 hours	^*∗*^ *P*-value	6 Months	^*∗*^ *P*-value
Activa BioActive	3.66 ± 0.50 **c**	0.0001	4.94 ± 0.16 **d**	0.0001	7.70 ± 0.73 **e**	0.0001
Cention N	3.98 ± 0.16 **c**		5.43 ± 0.17 **d**		8.44 ± 0.88 **e**	
RMGI	5.84 ± 0.50 **b**		4.68 ± 0.19 **bd**		5.18 ± 0.54 **b**	
Z250	0.07 ± 0.15 **a**		0.23 ± 0.27 **a**		0.10 ± 0.18 **a**	

^*∗*^One-way ANOVA. Similar lowercase letters show no difference according to Tukey's HSD test (*P*>0.05).

**Table 3:** Mean values of pH after 60 minutes of acidification of the sample environments (pH = 4.00 ± 0.01) in which the stored restorative materials (24 hours, 48 hours, and 6 months) were placed.

Materials	24 hours' stored samples	^*∗*^ *P*-value	48 hours' stored samples	^*∗*^ *P*-value	6 months' stored samples	^*∗*^ *P*-value
Activa BioActive	+4.28 ± 0.04 a	0.0001	+4.24 ± 0.02 a	0.0001	+4.53 ± 0.03 b	0.0001
Cention N	+4.39 ± 0.04 a		+4.29 ± 0.01 ac		+4.53 ± 0.04 b	
RMGI	+4.46 ± 0.04 a		+4.31 ± 0.04 ac		+4.66 ± 0.04 c	
Z250	+4.04 ± 0.01 d		+4.02 ± 0.00 d		+4.04 ± 0.00 d	

^*∗*^Two-way ANOVA. Similar lowercase letters show no difference according to Tukey's HSD test (*P*>0.05).

## Data Availability

The data are available upon request to the corresponding author.
